# Supplementary material for: MetaRibo-Seq measures translation in microbiomes
Source: Nat Commun. 2020 Jun 29;11:3268. doi: 10.1038/s41467-020-17081-z (PMC7324362; doi:10.1038/s41467-020-17081-z)
Supplement: Supplementary file 10 — Supplementary Data 7 [file 41467_2020_17081_MOESM10_ESM.zip › File2/Confidence_High_Taxonomy/174777_out.krona.html]

Javascript must be enabled to view this page.

members
magnitude
magnitudeUnassigned
count
unassigned
taxon
rank

174777\_out

2

2
superkingdom
2

1239
2
phylum

1
class
91061

1
order
186826

1300
1
family


SRS098057\_contig\_number\_8165
1
genus
1301

1
class
186801

order
1
186802


SRS147425\_contig\_number\_contig-100\_14699.54997
1
family
541000
